# Incorporating omics-based tools into endophytic fungal research

**DOI:** 10.1016/j.biotno.2023.12.006

**Published:** 2023-12-31

**Authors:** Vinita Verma, Alok Srivastava, Sanjay Kumar Garg, Vijay Pal Singh, Pankaj Kumar Arora

**Affiliations:** a Department of Environmental Microbiology, Babasaheb Bhimrao Ambedkar University, Lucknow, 226025, India; b Department of Plant Science, MJP Rohilkhand University, Bareilly, India

**Keywords:** Fungal endophytes, Bioactive compounds, Omics technologies

## Abstract

Fungal endophytes are valuable sources of bioactive compounds with diverse applications. The exploration of these compounds not only contributes to our understanding of ecological interactions but also holds promise for the development of novel products with agricultural, medicinal, and industrial significance. Continued exploration of fungal endophyte diversity and understanding the ecological roles of bioactive compounds present opportunities for new discoveries and applications. Omics techniques, which include genomics, transcriptomics, proteomics, and metabolomics, contribute to the discovery of novel bioactive compounds produced by fungal endophytes with their potential applications. The omics techniques play a critical role in unraveling the complex interactions between fungal endophytes and their host plants, providing valuable insights into the molecular mechanisms and potential applications of these relationships. This review provides an overview of how omics techniques contribute to the study of fungal endophytes.

## 1 Introduction

Endophytic fungi play a crucial role in plant health and protection. These organisms reside within the living, healthy tissues of plants without causing any visible disease symptoms.^1^^,^^2^ They establish themselves in specific sites within the plant, forming symbiotic relationships that do not harm the host.^3^ This colonization helps in preventing the entry and establishment of pathogenic microorganisms. By competing with pathogens for nutrients, endophytic fungi act as a barrier, limiting the resources available to potential plant diseases.^4^ This nutritional competition is a key factor in protecting plants from infections. Endophytic fungi are known to produce antibiotics, which can inhibit the growth of pathogenic microorganisms.^5^ This antibiotic synthesis is a form of defense that contributes to the overall health of the host plant. These fungi can induce resistance mechanisms within the plant, making it more resilient to various stresses and potential diseases.^6^ This proactive defense helps the plant withstand adverse conditions. Some endophytic fungi contribute to the growth of their host plants. They can produce phytohormones, which play a role in regulating plant development. This promotion of growth is beneficial for the overall vitality of the plant. Endophytic fungi enhance the plant's ability to resist different stresses, such as drought, salinity, or extreme temperatures.^7^ This adaptation contributes to the plant's survival and productivity in challenging environments. The endophytic fungus *Piriformospora indica* has been demonstrated to enhance the growth and development of host plant species under both normal and adverse conditions The role and impact of *P. indica* in the barley host plant under different extent of drought stress has also been reported.^8^ The *P. indica*, was observed to promote the biomass of both the roots and shoots of host plants. This positive effect is noted not only under conditions of scanty water but also in the presence of an adequate amount of water.

The fungal endophytes are the excellent source of multipurpose bioactive compounds.^9^ These metabolites play a diverse role in mitigating biotic and abiotic stress along with acting as antiviral, anti-carcinogenic and antimicrobial means.^10^ Genomics has been reported to provide a view of entire genomic level information of endophytic fungal isolates which help researchers to move towards the finding of novel genes coding for potent metabolic compounds which could be possible by merging genomics technology with metabolomics tool permitting to study various metabolites produced by fungal endophytes.^11^ On the other hand, metagenomics is also a significant technique in molecular biology domain that allows the direct analysis of entire genome within an environmental sample and insights relevant genome based data.^12^ Moreover, transcriptomics and proteomics technologies impart the profiling of gene expression and proteomes assembled by endophytic fungal strains, respectively.^13^ Thus, the implementation of omics is a novel approach to understand multi-functionality of fungal endophytes and their interaction with host plant thoroughly by the application of genomics, metagenomics, transcriptomics, proteomics and metabolomics means which could encourage researchers to discover more potent endophytic fungal isolates and advantageous bioactive compounds and also to know the complex strategies behind their interactions with host plant in order to explore them in distinct zones.

## 2 Role of omics tools in fungal endophytes

Omics technologies have played a pivotal role in advancing our understanding of endophytic fungi, providing insights into their genomics, transcriptomics, proteomics, metabolomics, and other molecular aspects. These technologies enable researchers to comprehensively study the genetic, metabolic, and functional attributes of endophytic fungi in various ecological and symbiotic contexts. In this section, role of various omics techniques are discussed.

### 2.1 Genomics

High-throughput sequencing technologies, such as next-generation sequencing (NGS) platforms like Illumina, PacBio, and Oxford Nanopore, have been instrumental in elucidating the genomic landscape of endophytic fungi. Whole-genome sequencing of fungal endophytes provides insights into their genetic makeup, potential for secondary metabolite production, and adaptations to the host environment. Comparing the genomes of different endophytes helps identify conserved genes, unique features, and potential virulence factors or symbiotic genes. Table 1 summarizes the list of fungal endophytes whose genomes have been sequenced. The member of genus *Alternaria* such as *Alternaria* sp. MG1 has its genome size 34.70 Mb.^14^ In a study conducted by Park et al. an endophytic fungal strain *Alternaria alternata* JS-1623 was isolated from the stem tissue of its host plant *Abies koreana*.^15^ Far from the phytopathogenic members of *Alternaria,* endophytic species of *Alternaria* have also been reported to produce many secondary metabolites; interestingly these metabolites are advantageous to medical domain due to their anti-inflammatory,^16^ anti-microbial,^16^^,^^17^ antiviral^18^ and anti-carcinogenic^19^ course of action. Few reports are there based on relevant genes encoding to produce secondary metabolites by endophytic fungal strain *Alternaria alternate* JS-1623. Keeping this aim in mind, Park et al.^15^ performed the whole genome sequencing of *Alternaria alternate* JS-1623 which resulted in an assembled genome size of 33.67 Mb. This outcome states WGS is a successful and powerful approach for the identification of genes biosynthesizing the potent secondary metabolites.

**Table 1:** A partial list of fungal endophyte for which their genomes have been sequenced.

S. No	Fungal Endophyte	Genome Size (Mb)	Accession No	Reference
1.	*Alternaria* sp. MG1	34.70	QPFE00000000	13
2.	*Amphirosellinia nigrospora* JS-1675	48.17	SCHM00000000	13
3.	*Ascocoryne sarcoides* NRRL 50072	34.00	AIAA00000000	13
4.	*Ascomycete* sp. F53	38.16	KT874412	13
5.	*Aspergillus versicolor* 0312	36.10	NA	13
6.	*Cadophora* sp. DSE1049	70.46	PCYN00000000	13
7.	*Calcarisporium arbuscula* NRRL 3705	45.01	WBSA00000000	13
8.	*Colletotrichum gloeosporioides* Cg01	55.77	QRFY00000000	13
9.	*Colletotrichum truncatum*	56.10	VUJX00000000	13
10.	*Dactylonectria torresensis* BV-349	64.42	VYKH00000000	13
11.	*Dactylonectria torresensis* BV-666	65.33	VYKG00000000	13
12.	*Diaporthe ampelina*	59.00	LWAD00000000	13
13.	*Diaporthe* sp. HANT25	55.33	JACBFG000000000	13
14.	*Fusarium solani* JS-169	45.80	NGZQ00000000	13
15.	*Fusarium tricinctum* T6	42.73	PTXX00000000	13
16.	*Gaeumannomyces* sp. strain JS-464	53.15	NGZR00000000a	13
17.	*Hypoxylon pulicicidum* MF5954	41.44	PDUJ00000000a	13
18.	*Hypoxylon* sp. CI-4A	37.70	MDGY00000000	13
19.	*Hypoxylon* sp. CO27	46.50	PDUJ00000000a	13
20.	*Hypoxylon* sp. E7406B	45.30	JYCQ00000000	13
21.	*Hypoxylon* sp. EC38	47.30	MDCK00000000	13
22.	*Metarhizium robertsii*	39.04	ADNJ00000000a	13
23.	*Mucor endophyticus* CBS 385–95	35.00	PRJEB30975	13
24.	*Penicillium aurantiogriseum* NRRL 62431	32.70	ALJY00000000	13
25.	*Penicillium brasilianum* LaBioMMi 136	32.90	LJBN01000000	13
26.	*Penicillium citrinum* DSM 1997	31.52	BCKA00000000	13
27.	*Penicillium polonicum* hy4	33.92	QPIC00000000	13
28.	*Periconia macrospinosa*	38.14	PCYO00000000	13
29.	*Pestalotiopsis fici*	52.00	ARNU00000000	13
30.	*Phialocephala scopiformis* DAOMC 229536	48.87	LKNI00000000	13
31.	*Phyllosticta capitalensis* CBS 128856 v1.0	32.46	1109085 (JGI)	13
32.	*Phyllosticta citribraziliensis* CBS100098	31.67	1109089 (JGI)	13
33.	*Pochonia chlamydosporia*	41.20	AOSW00000000	13
34.	*Purpureocillium lilacinum* TERIBC 1	38.82	LOFA00000000	13
35.	*Rhodotorula graminis* WP1	21.01	JTAO00000000	13
36.	*Sarocladium brachiariae*	31.86	RQPE00000000	13
37.	*Shiraia* sp. slf 14	32.06	AXZN00000000	13
38.	*Xylona heveae*	24.34	JXCS00000000	13

The fungal endophytes such as *Purpureocillium lilacinum* PLFJ-1 and *Purpureocillium lilacinum* TERIBC1 have been reported with their genome size of 38.53 Mb and 38.82 Mb, respectively.^20^ Moreover, the genome of *Hypoxylon* sp. E7406B (45.30 Mb),^21^ *Hypoxylon pulicicidum* MF5954 (41.44 Mb),^22^ *Hypoxylon* sp. CI-4A (37.70 Mb),^23^ *Hypoxylon* sp. EC38 (47.30 Mb)^23^ and *Hypoxylon* sp. CO27 (46.50 Mb)^23^ have also been sequenced.

The fungal endophyte *Simplicillium aogashimaense* strain 72–15.1 was originated from asymptomatic leaf of *Brachiaria brizantha*. The members of S*implicillium aogashimaense* are engrossing and crucial to economical and ecological point of view due to the production of a wide range of bioactive compounds^24^ and hence are used as bio-control agents.^25^ Jauregui et al. reported a high level of antifungal activity of S*implicillium aogashimaense* 72–15.1 against fungal phytopathogens such as *Bipolaris* sp. aff. *sorokiniana*, *Alternaria alternatum* and *Curvularia trifolii*.^26^ Moreover, the size of draft genome of S*implicillium aogashimaense* 72–15.1 sequenced resulted in 29 Mb.^26^

Further, the genome size of endophytic fungal strain *Fusarium tricinctum* T6^27^ was 42.73 Mb and *Fusarium solani*^28^ JS-169 was 45.80 Mb. Similarly, *Phyllosticta citribraziliensis* CBS100098 was reported with its genome size of 31.67 Mb whereas *Phyllosticta capitalensis* CBS128856 v1.0 with 32.46 Mb.^29^ Additionally, fungal isolate *Diaporthe ampelina*^30^ was reported with genome size of 59.00 Mb while *Diaporthe* sp. HANT25 has been found sequenced genome size of 55.33 Mb.^31^ Besides, the members of genus *Colletotrichum* such as *Colletotrichum gloeosporioides* Cg01^32^ and *Colletotrichum truncatum*^33^ have been found with their genome size of 55.77 Mb and 56.10 Mb respectively; on the other hand, members of genus *Dactylonectria* like *Dactylonectria torresensis* BV-349 and *Dactylonectria torresensis* BV-666 have been reported with the genome size of 64.42 Mb and 65.33 Mb, respectively.^34^

*Calcarisporium arbuscula* is a fungal endophyte of the fruit bodies of Russulaceae; have been reported to produce certain types of antibiotics and hence exhibits resistance towards other fungal strains.^35^ Interestingly, *Calcarisporium arbuscula* has been reported to produce various secondary metabolites that show antibiotic, anti-carcinogenic and anti-nematode activities. It is also capable of producing a huge amount of aurovertin-type mycotoxins acting as the inhibitor of F0F1-ATP synthase,^36^ including aurovertin B which is a potent therapeutic anti-carcinogenic agent^37^ whereas aurovertin D which is strongly affective against root knot nematodes, *Meloidogyne incognita*.^38^ These reports recommend for the utilization of *Calcarisporium arbuscula* as bio-control and in the development of therapeutic agent in medical terms. In a study conducted by Cheng et al.,^39^ identified sixty five gene clusters coding for the synthesis of secondary metabolites in endophytic fungal strain *Calcarisporium arbuscula* NRRL 3705 based on antiSMASH 4.0. Moreever, gene cluster for aurovertins synthesis was also identified in the same study. Additionally, various gene clusters were also predicted by the researchers in their investigation for the production of mycotoxins such as alternariol, citrinin, aflatoxin, isoflavipucine and destruxin.^39^ Moreover, out of sixty-five gene clusters twenty three were found to carry genes coding for the synthesis of PKS (polyketide synthases) while rest of twelve gene clusters contain genes encoding for NRPS (non-ribosomal peptides synthases) synthesis. Furthermore, assessment of gene expression was also done through FPKM (fragments per kilobase of exon per million mapped fragments) based RNA Sequencing. Based on housekeeping genes including *gpdA*, *tubC* and *actA* as reference genes; Cheng et al.^39^ further stated that only PKS (polyketide synthases) gene was reported for its expression at high extent out of all genes present in entire gene clusters identified in their research. These findings validate that majority of gene clusters are either silenced or expressed at low extent. Omics based data in addition to bioinformatics revealed that *Calcarisporium arbuscula* NRRL 3705 carries a wide number of biosynthetic gene clusters having potential to produce enormous secondary metabolites. The genome of *Calcarisporium arbuscula* NRRL 3705 was sequenced using high quality Single Molecule Real-Time (SMRT) sequencing technology. The genome of *Calcarisporium arbuscula* was assembled with an approximate size of 45.01 Mb. This genome has been reported to carry a vast number of gene clusters which play a key role in the biosynthesis of secondary metabolites such as aurovertins in addition to other mycotoxins.^39^ Thus, genome related data can be applicable in comparative genomics based analysis and to discover novel genes in fungal endophytes encoding for additional potent secondary metabolites in future trends using omics tool.

The genome of various members of the genus *Penicillium* for instance, *Penicillium brasilianum* LaBioMMi 136 (32.90 Mb),^40^ *Penicillium citrinum* DSM 1997 (31.52 Mb),^41^ *Penicillium aurantiogriseum* NRRL 62431 (32.70 Mb)^42^ and *Penicillium polonicum* hy4 (33.92 Mb)^32^ have been sequenced. In addition, *Colletotrichum gloeosporioides* Cg01 and *Penicillium polonicum* hy4 fungal endophytes of *Huperzia serrata* have been reported to produce Huperzine A^32^. Furthermore, *Shiraia* sp. Slf14 a fungal endophyte of *Huperzia serrata* has also been reported to produce Huperzine A.^43^ Moreover, an endophytic fungal strain *Penicillium aurantiogriseum* NRRL 62431 isolated from *Corylus avellana* has been reported to produce Taxol (paclitaxel).^42^ Similarly, few members of genus *Aspergillus* including *Aspergillus nidulans,*^44^ *Aspergillus niger,*^45^ *Aspergillus versicolor,* 0312^46^ and *Aspergillus oryzae*^47^ have been reported with their genome size of 30.10 Mb, 33.90 Mb, 36.10 Mb and 36.70 Mb respectively. Additionally, *Harpophora oryzae* an endophyte of *Oryza granulate* and *Oryza sativa* has been reported to produce Indole derivatives.^48^ The endophyte *Pestalotiopsis fici* in tea plants is reported to produce various secondary metabolites, including pestalofones, chloropupukeananin, pestaloficiol, chloropestolides, chloropupukeanolides, pestalodiols, and chloropupukeanone.^17^ These secondary metabolites play a crucial role in exhibiting several biological activities, including: Prevention of tumor cytotoxicity, Inhibition of HIV-1 replication. Acting as anti-fungal agents. The genome-based analysis of *Pestalotiopsis fici* revealed abundance of carbohydrate-active enzymes, particularly pectinases and identified a significant number of genes capable of synthesizing secondary metabolites are identified. The abundance of genes involved in the synthesis of secondary metabolites indicates its potential to produce natural products with various biological activities.

Integrated multi-omics techniques were used to study *Ascocoryne sarcoides*, a endophyte of *Picea mariana*. The focus was on understanding the production of C8 volatiles, an uncommon secondary metabolite.^49^ The whole-genome sequencing of *Penicillium aurantiogriseum* were employed to characterize genes.^42^ This led to the synthesis of paclitaxel, demonstrating independence from the host plant's biosynthetic pathway. Genome analysis of *Epicoccum nigrum* revealed the endophyte's ability to enhance sugarcane root biomass.^50^ Metabolic biocontrol agents were formed to protect against various plant pathogens of sugarcane. The genome sequence of fungal endophyte *Gaeumannomyces* sp. in *Phragmites communis* demonstrated the production of secondary metabolites with anti-inciting activities.^51^

Overall, genomic studies contribute to our understanding of the diverse roles that fungal endophytes play in plant ecosystems and agriculture. They highlight the potential applications of these fungi in various fields, from medicine (e.g., paclitaxel synthesis) to agriculture (e.g., biocontrol agents, plant growth promotion).

### 2.2 Transcriptomics

Transcriptomics is a branch of molecular biology that involves the study of an organism's transcriptome, which comprises all the RNA molecules produced by the genome at any given time.^52^ The primary focus of transcriptomics is on analyzing and understanding the transcript-level expression of genes, including the types and abundance of RNA molecules, such as messenger RNA (mRNA), non-coding RNA (ncRNA), and small RNA.^52^

RNA-Seq is a widely used technique in transcriptomics.^52^ RNA Sequencing (RNA-Seq) is used to analyze the entire transcriptome of the fungal endophyte. It provides information about gene expression levels, alternative splicing, and novel transcripts under different conditions, helping researchers understand the dynamic changes in gene expression during the interaction with the host.

Microarray technology is another method for transcriptomic analysis.^53^ Microarrays allow researchers to measure the expression levels of thousands of genes simultaneously.^53^ Transcriptomics helps identify genes that are upregulated or downregulated under specific conditions or in response to stimuli. Differential gene expression analysis is crucial for understanding how gene expression patterns change in different biological states.^53^

One of the earliest transcriptome-based reports focused on analyzing genes associated with pyrimidine metabolism in *Epulorhiza* sp. a fungal endophyte isolated from *Anoectochilus roxburghii,* an orchid's roots.^54^ The significantly differentially expressed antifungal genes in the endophytic fungus *Epichloë festucae* integrated with the grass *Festuca rubra* has been reported.^55^ This highlights the molecular interactions and gene expression changes in the endophyte-grass symbiotic relationship. Integrated omics approaches (genomics, transcriptomics, and metabolomics) were used to analyze genes associated with cellulose biodegradation and biofuel production in the fungal endophyte *Ascocoryne sarcoides*.^49^ Shaffer et al.^56^ developed a methodology to evaluate gene expression in both endohyphal *Luteibacter* sp. and endophytic fungal host *Pestalotiopsis* sp. when grown individually and in co-culture. Dual RNA-sequencing data revealed that co-culturing the bacterial and fungal strains regulates metabolic and developmental processes in both organisms.^56^ The regulation may involve alterations in the equilibrium of organic sulphur through methionine acquisition.

The transcriptome of the metal-tolerant fungal endophyte *Exophiala pisciphila* was analyzed in the presence of different heavy metal residues.^57^ Several glutathione S-transferase genes were examined to understand the response to heavy metal stress.^57^ Differential gene expression analysis was performed on the fungal endophyte *Trichoderma virens* isolated from *Zea mays* using the NovaSeq 6000 sequencing platform.^58^ HiSeq 2500 sequencing system was used to identify the expression of genes involved in detoxification, hydrolytic enzyme synthesis, and redox homeostasis in the fungal endophyte *Serendipita vermifera* of *Hordeum vulgare*.^59^ Strains of *Epichloë coenophiala* isolated from *Festuca arundinacea* were studied for differential gene expression using Illumina HiSeq 2000 and HiSeq 2500 platforms.^60^ Differential gene expression related to flavonoid biosynthesis by *Ceratobasidium* sp. AR2 isolated from *Anoectochilus roxburghii* was reported via Illumina HiSeq 2000 sequencing.^61^ The fungal endophyte *Serendipita indica* of *Zea mays* was studied for differential gene expression involved in the biosynthesis of auxin, suberin, wax, cutin, and signalling pathways using the Illumina HiSeq system.^62^ The Illumina HiSeqTM 4000 sequencing platform was used to analyze the expression of genes responsible for primary metabolism and terpene skeleton biosynthesis in the fungal endophyte *Gilmaniella* sp. AL12 of *Atractylodes lancea*.^63^ HiSeq machines provided data on differential gene expression in *Trichoderma asperellum* related to plant physiology, photosynthesis, root development, stomatal and RNA activity, and overall plant growth promotion.^64^

### 2.3 Proteomics

Proteomics is the study of the entire set of proteins produced by an organism, tissue, or cell at a particular time.^65^ Proteomics can be used to identify the proteins produced by fungal endophytes. It involves the identification, characterization, and quantification of proteins to understand their functions and interactions. The integration of 2D-gel electrophoresis and mass spectrometry is indeed a widely accepted approach in proteomics.^65^ Currently, Mass Spectrometry based proteomics technique is a most powerful approach for mapping the complete set of proteins produced by fungal endophytes which insights the better understanding of their interactions with host plants including biological pathways as well as post translational modifications.^66^ In particular, an entire set of protein in *Atractylodes lancea* inoculated with and without endophytic fungal strain *Gilmaniella* sp. AL12 was reported in a study conducted by Yuan et al.^63^ Moreover, they also found the upregulation of proteins associated with carbon fixation as well as carbohydrate & energy metabolism in their investigation that suggests endophyte *Gilmaniella* sp. AL12 could promote biomass of *Atractylodes lancea*.^63^

Hilário and Gonçalves^67^ focused on proteomic research on the members of filamentous fungal endophyte genus *Diaporthe* and stated that there is no proteomics-based reports in this fungi. As proteome profiling permits researchers for structural and functional based assessment of dynamic proteins within an organism under specified condition, therefore, proteomics based research on the mechanism behind the interaction of endophytic *Diaporthe* strains with their host plants should be done thoroughly.

The mass spectrometry for proteome analysis was used in *Piriformospora indica* to characterize various proteins involved in different mechanisms such as reactive oxygen species (ROS) defence, photosynthesis, and energy transport under drought conditions. However, limited information obtained solely through proteomics-based studies underscores the complexity of plant-fungal endophyte interactions. Integrating multiple omics techniques can overcome this limitation by capturing a broader range of molecular information.

### 2.4 Metagenomics

**Fig. 1:**
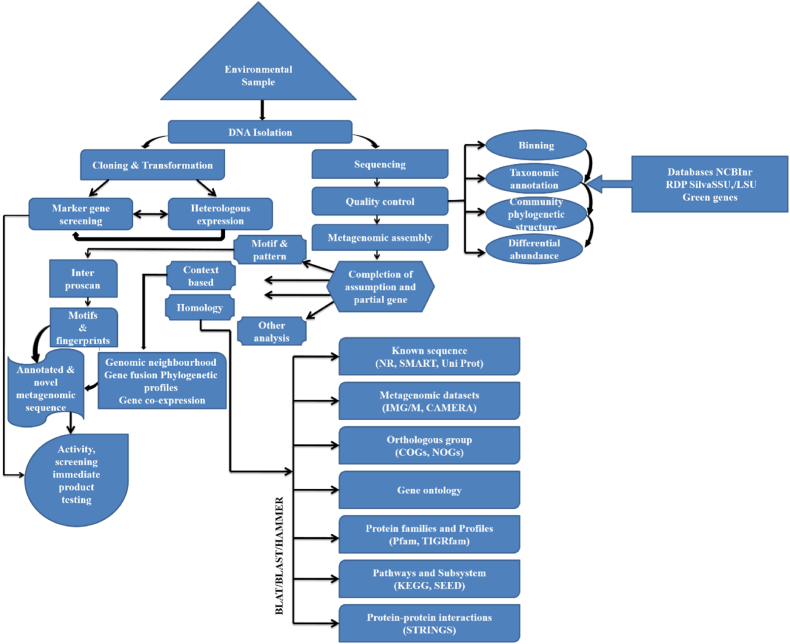
Metagenomic analysis of fungal endophytes^68^

Metagenomics plays a crucial role in the study of fungal endophytes, providing a powerful tool to explore the diversity, functional potential, and ecological roles of these fungi within plant hosts and their associated environments.^68^ Metagenomics allows for the exploration of fungal diversity within plant tissues and other environmental samples. It enables the identification of various fungal species and their abundance, offering insights into the composition of fungal endophyte communities. High-throughput metagenomic technologies enable a deep understanding of microbial structure, function, and community dynamics (Fig. 1). These technologies have been applied in screening fungal endophytic strains. Preliminary analyses of fungal communities were traditionally culture-based, and ITS region sequencing was applied, but it was limited to a deficient number of samples. Furthermore, a study based on Internal Transcribed Spacer (ITS) region sequencing was conducted in order to identify a fungal endophyte isolated from the medicinal tree viz. *Aquilaria malaccensis* which proposed a mass findings on it.^69^ A revised protocol for an automated method of ribosomal intergenic spacer analysis (ARISA) was developed to diagnose fungal diversity in environmental samples. This targeted polymorphisms in the ITS1 rDNA length of fungal species.^70^ A first report based on employing next generation sequencing (NGS) to analyze the ITS region of residential endophytic species in a host plant from South Africa was brought out in the year 2013.^71^ To present a network representing figures for the co-occurrence of symbiosis, the application of Illumina sequencing of fungal species engaged with mono dominant forest roots was stated in another study.^72^

Currently, metagenomics along with computational tools is a considerable molecular approach for functional gene identification in fungal endophytes from their host plants. *Ephedra sinica* is a model plant to study the relationship between fungal endophytes and different tissues of the host plant showing distinct biological potencies. Metagenomic and metabolomic based systematic study on the community of fungal endophytes and their relevant metabolic compounds in the roots and stem of *Ephedra sinica* was conducted by Zhang et al.^73^ Results revealed that fungal strains OTU30589 and OTU48 were the endophytes of roots and stem respectively. Moreover, a specific and abundant occurrence of endophytic fungal strains of the genus *Phyllosticta* was reported in the stem of *Ephedra sinica* while a huge number of endophytic members of the genera *Aspergillus*, *Talaromyces* and *Aporospora* were present in the roots of the same.^73^

### 2.5 Metabolomics

The study of metabolomics in the context of endophytic fungi involves analyzing and understanding the complete set of small molecules (metabolites) produced by these fungi. Metabolomics provides a powerful approach to explore the metabolic profile of these fungi and their interactions with host plants.^74^ Metabolomics allows for a comprehensive profiling of the metabolites produced by endophytic fungi. This includes the identification and quantification of small molecules such as secondary metabolites, amino acids, organic acids, and other bioactive compounds. Nuclear Magnetic Resonance (NMR) spectroscopy and mass spectrometry are commonly used techniques to study the diverse array of secondary metabolites and other bioactive compounds synthesized by these fungi.^74^ Metabolomics is instrumental in identifying and characterizing secondary metabolites, contributing to the discovery of potential pharmaceutical or agricultural applications. Table 2 presents a list of some of the secondary metabolites (bioactive compounds) produced by fungal endophytes.

**Table 2:** Bioactive compounds produced by fungal endophytes.

Fungal endophyte	Host Plant	Compound	Activity	Reference
*Pestalotiopsis terminaliae*	*Terminalia arjuna*	Paclitaxel	Induction of apoptosis	54
*Alternaria* sp.	*Crotalaria pallida*	Coumarin	Caspase-dependent apoptosis induction	54
*Ascomycetes* sp.	*Mimusops elengi*	Ergoflavin	Cytotoxicity induction, Anti-inflammatory action	54
*Cephalotheca faveolata*	*Eugenia jambolana* Lam.	Sclerotiorin	Regulates apoptosis	54
*Botryodiplodia theobromae*	*Morinda citrifolia* Linn.	Paclitaxel	COX-2 downregulation, Re-establishment of enzyme based anti-peroxidative activity	54
*Talaromyces wortmannii*	*Aloe vera*	Component C	Antibacterial activity against *Propionibacterium acnes*, Anti-inflaming activity	54
*Pestalotiopsis microspora*	*Terminalia morobensis*	Isopestacin	Quenching OH and superoxide O_2_ radicals, Anti-fungal performance against *Sclerotinia sclerotioru, Rhizoctonia solania and Pythium ultimum*	54
*Alternaria* sp.	*Viscum album*	Lectin (N-acetyl galactosamine)	Suppression of sucrose, α-glucosidase and α-amylase; as well as the induction of ductal stem cells	54
*Phoma* sp.; *Aspergillus* sp. JPY1 and *Aspergillus* sp. JPY2	*Salvadora oleoides*	Phenol, 2, 6-bis (1,1-dimethylethyl)-4-Methyl; 2, 6-di-*tert*-butyl-p-cresol	Renewal of pancreatic cells and enhancing beta cells mediated insulin secretion	54
*Lasiodiplodia theobromae*	*Vitex pinnata*	Desmethyllasiodiplodin, R-(−)- mullein Cladospirone B	Anti-parasitic activity inopposition to *Trypanosoma* spp.	54
*Emericella* sp.	*Aegiceras corniculatum*	Asper-nidine, austinol, austin, acetoxydehydroaustin, Emerimidine, emeriphenolicins and dehydroaustin	Antiviral activity in opposition to Influenza A (H1N1) virus	54
*Cytonaema* sp.	*Quercus* sp.	Cytonic acid	Antiviral activity in opposition to human cytomegalovirus and Hepatitis virus	54
*Alternaria tenuissima*	*Quercus emoryi*	Altertoxin I, II, III and V	Antiviral activity in opposition to HIV-1 virus	54
*Periconia atropurpurea*	*Xylopia aromatica*	3′-dienyl]-Benzaldehyde, 2,4-dihydroxy-6-[(1′E,3′E)-penta-1′,	Anti-fungal activity in the opposition of *C. Cladosporioides, Cladosporium sphaerospermum,*	54
*Aspergillus fumigatus*	*Astragalus membranaceus*	Verruculogen, Cyclotryprostatin B, Fumitremorgin B, C, Gliotoxin Cyclotryprostatin C,	Performs anti-fungal role against *Fusarium solani, P. chrysogenum and C. albicans,*	54
*Eurotium cristatum* EN-220	*Sargassum thunbergii*	Indole diketopiperazine alkaloids	Nematocidal activity against *Panagrellus redivivus*	54
*Xylaria grammica*	*Menegazzia* sp.	Grammicin	Nematocidal activity against *Meloidogyne incognita*	54
*Melanconium betulinum*	*Betula. pubescens, B. pendula*	3-Hydroxy propionic acid	Nematocidal activity against *Meloidogyne incognita*	54
*Aspergillus fumigatus* JRJ111048	*Acrostichum speciosum*	Anhydride derivative aspergide	Insecticidal activity against *Spodoptera litura*	54
*Berkleasmium* sp.	*Dioscorea zingiberensis*	Spirobisnaphthalenes	Insecticidal activity against *Aedes albopictus*	54
*Penicillium oxalicum* LA-1	*Limonia acidissima* L.	Hamisonine	Insecticidal activity against *Culex quinquefasciatus*	54
*Pichia guilliermondii*	*Paris polyphylla* var. *yunnanensis*	helvolic acid and 5α,8α epidioxyergosta-6,22-dien-3β-ol	Antibacterial activity against *R. solanacearum, E. coli, P. lachrymans, S. aureus, S. haemolyticus, X. Vesicatoria, A. Tumefaciens, B. subtilis*	54
*Aspergillus fumigatus*	*Astragalus membranaceus*	Fumiquinazoline C, D and J, Cyclotryprostatin B, Fumitremorgin B and C, Verruculogen, Gliotoxin, Methylthiogliotoxin, Cyclotryprostatin C,	Antibacterial activity against *E. coli, B. subtilis, P. aeruginosa S. aureus*	54
*Syncephalastrum* sp.	*Adathoda beddomei*	Phenyl esters, Furandione, diethyl phthalate, anthracene methanol, pentadecanoic acid	Antibacterial activity against *S.* Aureus, *Klebsiella pneumoniae, Escherichia coli, Pseudomonas aeruginosa, C. albicans*	54

Recently, Nagarajan et al. reviewed recent development of metabolomics studies on endophytic fungi.^74^ A metabolomic-based comparative analysis of the fungal endophyte *Aspergillus terreus* isolated from *Opuntia ficus-indica* was carried out.^75^ Eleven different cultural conditions were employed to grow the fungus. The study identified a novel compound, 7-desmethylcitreoviridin, along with sixteen distinct fungal endophytic metabolic compounds.^75^ The approach allowed for a comparative analysis of metabolite production under different cultural conditions. Another metabolomics study aimed to investigate novel bioactive compounds produced by fungal endophytes. LC-MS (Liquid Chromatography-Mass Spectrometry) was employed as the analytical method. *Geomyces* sp., an endophytic fungus isolated from the plant Nerium indicum, was found to produce vincamine.^76^ A separate LC-MS-based metabolomics study focused on vincamine, a nootropic drug, and its analogue.

Metabolomics has been applied to study the effects of *Trichoderma* species on plant growth. Auxins produced by *Trichoderma* have been reported to promote plant growth by mitigating the deleterious impacts of stress.^77^ Secondary metabolites like 6-pentyl-α-pyrone and harzianolide produced by *Trichoderma* contribute to the growth and development of plants, similar to auxins.^78^ Metabolomics facilitates the study of how various environmental factors influence plant metabolism, leading to changes in the secretion of compounds and, consequently, affecting plant-microbe interactions.

## 3 Conclusions

The rapid development of omics techniques contributes to gaining deeper knowledge about the mechanisms of endophytic fungal strains and their impacts on host plants. Integration of genetics, metagenomics, and other omics tools is crucial for unraveling the complex interactions between endophytic fungal flora and their associated host plants. Omics techniques provide extensive information about the metabolic strategies, gene cascades, and the accumulation of metabolites, enzymes, and proteins in endophytic fungal communities.

Understanding the up and down-regulating systems of distinct genes is essential for deciphering the complexity of these interactions. Multi-omics approaches play a vital role in analyzing the mutualistic interaction of fungal endophytes with their host plant communities.

Transcriptomics approaches, while informative, may have limitations in sequencing data coverage, but re-sequencing and analytical techniques offer opportunities for improved assemblies. The creation of well-defined integrated databases from multi-omics research can enhance our understanding of endophytic fungal species. System biology approaches, along with integrated omics data, can be beneficial for prognostic modeling and dynamic evaluation of fungal endophytes with other microbial communities.

## Availability of data and material

Not applicable.

## Funding

No funds is available for this article.

## Authors' contributions

All authors contributed in the manuscript. All authors read and approved the manuscript.

## Declaration of competing interest

The authors declare that they have no known competing financial interests or personal relationships that could have appeared to influence the work reported in this paper.
